# Pilot study of an interactive voice response system to improve medication refill compliance

**DOI:** 10.1186/1472-6947-8-46

**Published:** 2008-10-09

**Authors:** Kristen Reidel, Robyn Tamblyn, Vaishali Patel, Allen Huang

**Affiliations:** 1 Clinical and Health Informatics Research Group, McGill University, 1140 Pine Avenue West, Montreal, Quebec, H3A 1A3, Canada; 2 Department of Epidemiology, Biostatistics, and Occupational Health, McGill University, 1040 Pine Avenue West, Montreal, Quebec, H3A 1A2, Canada; 3 Division of Geriatric Medicine, McGill University Health Centre, Royal Victoria Hospital, 687 Pine Avenue West Room M8.12, Montreal, Quebec, H3A 1A1, Canada; 4 Department of Pediatrics, Weill Cornell Medical College, 411 E.69th Street, NY, NY 10021, USA

## Abstract

**Background:**

Sub-optimal adherence to prescribed medications is well documented. Barriers to medication adherence include medication side effects, cost, and forgetting to take or refill medications. Interactive Voice Response (IVR) systems show promise as a tool for reminding individuals to take or refill medications. This pilot study evaluated the feasibility and acceptability of using an IVR system for prescription refill and daily medication reminders. We tested two novel features: personalized, medication-specific reminder messages and communication via voice recognition.

**Methods:**

Patients enrolled in a study of electronic prescribing and medication management in Quebec, Canada who were taking chronic disease-related drugs were eligible to participate. Consenting patients had their demographic, telephone, and medication information transferred to an IVR system, which telephoned patients to remind them to take mediations and/or refill their prescriptions. Facilitators and barriers of the IVR system use and acceptability of the IVR system were assessed through a structured survey and open-ended questions administered by telephone interview.

**Results:**

Of the 528 eligible patients who were contacted, 237 refused and 291 consented; 99 participants had started the pilot study when it was terminated because of physician and participant complaints. Thirty-eight participants completed the follow-up interview. The majority found the IVR system's voice acceptable, and did not have problems setting up the time and location of reminder calls. However, many participants experienced technical problems when called for reminders, such as incorrect time of calls and voice recognition difficulties. In addition, most participants had already refilled their prescriptions when they received the reminder calls, reporting that they did not have difficulties remembering to refill prescriptions on their own. Also, participants were not receptive to speaking to an automated voice system.

**Conclusion:**

IVR systems designed to improve medication compliance must address key technical and performance issues and target those individuals with reported memory difficulties or complex medication regimens in order to improve the utility of the system. Future research should also identify characteristics of medication users who are more likely to be receptive to IVR technology.

## Background

Sub-optimal adherence to prescribed medications is well documented and has significant consequences for patient health and care delivery [1-4]. On average, only half of all prescribed medications are consumed by the patient [1]. In particular, poor medication adherence results in increased utilization of health care resources and costs [5]. Barriers to medication adherence include medication side effects [5], cost [5,6], and forgetting to take or refill medications [7]. Many interventions to improve medication adherence have been targeted at reminding patients to take their pills or refill prescriptions on time. However, a review of interventions to improve medication adherence in the elderly found that the use of reminders on medication packaging [8], reminder calendars[8], and mailed reminders [9], did not did not improve medication adherence over control groups. This review concluded that telephone-based interventions showed the most promise in improving medication adherence [8].

Interactive Voice Response (IVR) systems are a type of computer-linked telephone intervention system that could be used to provide efficient reminders to refill medication. IVR can provide individualized messages to participants and obtain feedback from participants' responses through voice recognition or touchtone keypad. IVR systems have shown potential for use as a tool in health care. For example, IVR has been studied for use as a reminder system to increase preventive screening and vaccinations [10], as a means for screening adults, adolescents, and high-risk pregnant women for depression [11-13], for screening older adults for early signs of dementia [14,15], for measuring drinking levels as part of an alcohol treatment program [16], and for preventing drinking relapse in substance-abuse patients [17]. Generally these studies have shown that patients can and will use IVR systems [12,16], that the information collected using IVR systems is reliable and valid when compared to paper-based collection of information [11,13,15], and, in some studies, that the use of IVR has had a desired, positive effect on participants' behaviour [10,18].

Despite these potential benefits, there have been few attempts to use IVR to improve medication adherence [19]. One promising study used a dial-in service to support self-management of hypertension [20]. Participants phoned the service weekly to report medication adherence, side effects, and blood pressure, and they received educational messages regarding the benefits of medication adherence. The results showed increased adherence and decreased blood pressure amongst those who received the intervention and were not considered adherent to medications at baseline [20]. However, because it was up to the patients to call in to the system, the patients who were most likely to call were probably the most likely to benefit from the intervention. In addition, this type of single-disease intervention is not well suited to individuals with multiple chronic conditions taking many medications.

One pilot study of 16 older adults tested the feasibility of using IVR to improve medication adherence by asking subjects to follow a complex, but hypothetical, medication regime [7]. Subjects were required to scan barcodes of the pills they were scheduled to take at the times indicated by the medication schedule. Those subjects who received telephone reminders to scan their bar-coded medications showed a significant improvement in their ability to follow the simulated pill-taking instructions [7]. These results from an IVR intervention tested in simulated medication regimens are promising, but the IVR medication reminders have not yet been validated in actual practice. Additionally, more advanced methods such as voice recognition, which may increase accessibility for those with limited manual dexterity or visual impairment, have not been studied.

The purpose of this pilot study was to evaluate the feasibility and acceptability of an IVR system for prescription refill and daily medication reminders. This IVR system provided two features not previously tested: personalized, medication-specific reminder messages communicated to participants currently on medications, and communication via voice recognition.

## Methods

### Context

The source population for this study was 13,278 patients enrolled in the Medical Office of the 21^st ^century program (MOXXI) as of April 1^st ^2004. This ongoing research program is evaluating the implementation and persistent use of an electronic prescribing and medication management application in a group of physicians based in a large urban setting in Quebec, Canada. Participating physicians obtain consent from their patients to access their personal health information through the MOXXI system, including their participation in various research projects aimed at improving the safety and effectiveness of drug management.

Unique to the MOXXI system is its real-time link with the provincial health services insurance databases at la Régie de l'assurance Maladie du Québec (RAMQ) which includes the complete record of dispensed prescriptions for all those registered with the public drug insurance plan in Quebec, approximately 50% of the Quebec population [21]. This feature enabled the implementation of an IVR system for medication refill reminders since all patients participating in the MOXXI program who were covered by the public drug insurance plan had their complete medication profile readily available via the MOXXI system, including the dispensing dates of prescriptions written by all physicians for a particular patient.

### Sample Selection

The current medications for all patients enrolled in the MOXXI program were examined for the presence of drugs used to treat cardiovascular, diabetes, thyroid and respiratory diseases. These drugs were targeted as patients generally take these medications for extended periods of time, presenting opportunity for improving medication adherence. Patients were deemed eligible to participate in the pilot if they were dispensed at least one of these medications in the three months prior to the start of the pilot study (April 2004). To be included patients were also required to be publicly insured by the RAMQ in order to ensure their prescription drug information was complete. No other inclusion/exclusion criteria were used. Prior to initializing the intervention, eligible MOXXI patients were called to verify their interest in participating and to advise them about the start of the intervention. All patients who confirmed their consent then had their demographic, telephone, and medication information transferred to the IVR system to conduct the initial registration process.

### Intervention

An initial call was made by the IVR system to participants to set up their individual preferences and to record their voiceprint for identification purposes. Patients had the option to choose the time of day when the IVR calls would be made, what phone number should be called, and whether they wanted daily medication reminders and prescription refill reminders, or just prescription refill reminders. The IVR system would then ask them to say their name in order to record it for identification purposes in subsequent calls. After initial set-up, daily updates were provided to the IVR system on current medications for each patient, including drug identification number and name, and start and expected end-dates of current prescriptions. The IVR system made calls to remind patients to take mediations and/or refill their prescriptions according to the preferences chosen by the participant and their dispensed medication refill due dates. Reminder calls for refilling prescriptions were made three days before the end of participants' current prescription in order to give participants enough time to get to the pharmacy for their refill. Participants also had the option to have their prescription refill request sent directly to the pharmacy, and could also have the prescription delivered to their home. Participants were free to call in to the system at any time to change their preferences, temporarily stop the calls, or withdraw from the program.

The IVR system used VoiceXML a special markup language designed to facilitate the creation of interactive voice response (IVR) services. It enabled the playing of speech prompts using pre-recorded and text-to-speech information, accepting spoken commands (via speech recognition) and the recording of caller audio information. Following informed consent, the web server of the IVR supplier (Tagge Medical Systems) was sent the patient name, and their preferred telephone number. Once a patient was registered in the IVR system, daily updates of the medication(s) were sent to the IVR webserver. IVR calls, interaction, and data collection were automated by VoiceXML and pre-recorded scripts. The IVR system was tested multiple times in-house by research staff prior to pilot testing. Data obtained from the IVR system was extracted and analysed by the research team.

### Participant Feedback

Acceptability of the IVR system among participants was assessed through a structured survey and open-ended questions administered by telephone interview to identify facilitators and barriers of the IVR system use. Participants were asked about technical aspects of the system, such as clarity and volume of the IVR voice, whether the calls were made at the times requested, and whether the system identified the correct medications to be reminded of or to be refilled. Participants were also asked for feedback regarding their understanding of the purpose of the calls and the perceived usefulness of the IVR system for helping them to remember to take/refill their medications.

### Analysis

Descriptive statistics were used to summarize and compare demographic and medication characteristics of participants who completed the follow-up interview to those who did not complete the follow-up interview, and also to compare those who reported they found the intervention helpful to those who did not. The open-ended interview questions provided opportunities for participants to elaborate on issues raised in the structured survey and voice their thoughts about the acceptability of the IVR system. Participants' comments were analyzed using qualitative data analysis techniques. This analysis involved categorizing responses to identify key aspects of the intervention's design and implementation that affected participants' perceptions of the overall utility of the system. Key facilitators and barriers were identified by linking participants' comments to their survey responses using a mixed method approach to integrate the qualitative and quantitative data [22].

## Results

**Figure 1:**
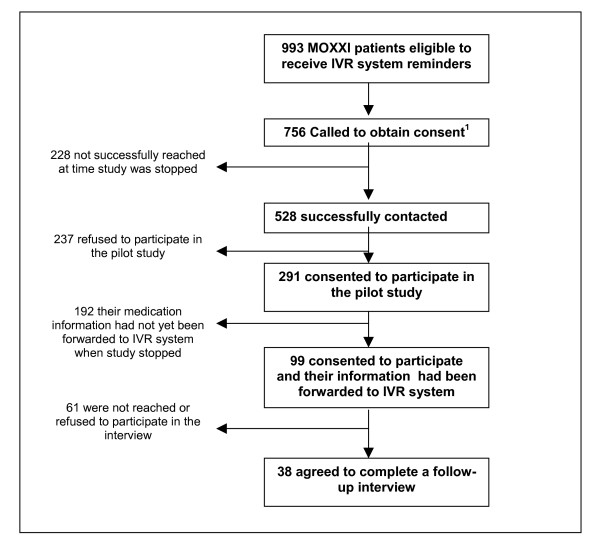
**Study population**. 1. 756 of the 993 had been called at the time when the pilot study was ended.

At the start of the study, 756 patients were eligible to participate, of which 228 (30.2%) could not be reached due to wrong numbers or no response upon repeated calls. Of the 528 successfully contacted, 291 (55.1%) agreed to participate in the pilot study and 237 (44.9%) refused. Of those who agreed to participate, the first 99 (34.0%) enrolled had their information sent to the IVR system (Figure 1), and the first IVR call was made on April 17^th^, 2004. However, the pilot study terminated prematurely after two months because of physician and participant complaints. The follow-up interviews were conducted with the first patients enrolled to evaluate the system.

Of the 99 participants in the pilot study, approximately half were female (Table 1). Participants tended to be older, with over 70% of participants being 70 years of age or greater. The majority of participants (72.7%) spoke French as their first language. On average, each participant had 16 medications dispensed in the first month of the study (April 2004), approximately 6 of which were for the target chronic disease medications. A follow-up interview was successfully completed with 38 of the initial 99 participants (38.4%). Participants of the follow-up interview were more likely to be female, age 80 years or older, and to speak English as their first language, however the differences in age, gender, and language distributions were not great (Table 1). Follow-up interview participants also had a greater number of active prescriptions than non-participants, both when considering all types of drugs and when considering targeted drugs for chronic diseases.

**Table 1:** Characteristics of the 99 patients who participated in the IVR pilot study

	**Participated in follow-up survey:**					
	**Yes (N = 38)**		**No (N = 61)**		**All (N = 99)**	
	**N**	**%**	**N**	**%**	**N**	**%**
**Female**	20	52.6	27	44.3	47	47.5
**Male**	18	47.4	34	55.7	52	52.5
						
**< 60 yrs**	2	5.3	5	8.2	7	7.1
**60–69 yrs**	7	18.4	14	23.0	21	21.2
**70–79 yrs**	17	44.7	28	45.9	45	45.5
**> 80 yrs**	12	31.6	14	23.0	26	26.3
						
**Primary language:**						
**French**	24	63.2	48	78.7	72	72.7
**English**	14	36.8	13	21.3	27	27.3
						
	**Mean**	**SD**	**Mean**	**SD**	**Mean**	**SD**
						
**Active prescriptions**	16.4	14.1	15.9	13.0	16.1	13.3
**Active prescriptions for chronic disease targeted by study**	5.2	3.8	5.9	4.8	5.6	4.4

### Participant understanding of the purpose of the IVR system

The majority of survey participants experienced some confusion regarding the purpose of the initial IVR system call. Twenty-one (57.6%) participants responded that they did not understand what the call was about the first time the IVR system contacted them (Table 2). Participants made comments such as, "I did not understand anything; it was confusing", "I didn't know what this was about at first" and "I did not really understand what it was about because I was not expecting this. It had been a long time since I had signed up for the program." In contrast to these individuals, those who reported understanding the purpose of the IVR system phone calls said that either their physician had explained the pilot study to them or provided them with adequate information about the pilot study.

**Table 2:** Participants' opinion regarding the set-up, timing, and accuracy of reminder calls

	***Survey Responses:***					
	**Yes**		**No**		**Don't Remember**	
***Interview questions:***	**N**	**%**	**N**	**%**	**N**	**%**
A. Did you understand what the automated telephone call was about?	17	44.7	21	55.3		
B. Did you receive a reminder call from the IVR system?	28	73.7	10	26.3		
*The following questions pertain only to those who answered yes to having received a reminder call (n = 28):*						
C. Did you have any difficulty setting up the time and location of the reminder call?	4	14.3	24	85.7		
D. Did the reminder call come in at the time you requested?	8	29.0	6	21.0	14	50.0
E. Was the name of the medication(s) to be refilled correct?^1^	18	78.3	0	0.0	5	21.7
F. When the reminder call came, had you already refilled your prescription?	20	71.4	8	28.6		
G. Did you find the call system helpful for remembering to refill your prescription?	4	14.3	24	85.7		

^1^5 participants did not answer this question

### IVR system technical performance and design

The majority of survey participants (89.5%) found the clarity of the IVR system's voice to be good or fair, and 34 (87.9%) found the volume of the IVR voice to be acceptable. In the open-ended interview, only a few participants made comments that "the words were not very clear or distinct".

The system ran relatively smoothly with regards to setting up the time and location of reminder calls. Among the 28 (73.7%) who had received a call from the IVR system at the time of the interview, 24 (85.7%) reported that they did not have trouble setting up the time and location of their reminder calls.

However, there were many major technical and performance issues reported by participants. Only 6 (21.0%) participants responded that the calls came at the time of day they had requested. Many patients interviewed mentioned they received calls too late in the evening; participants commented "I got a call at 12:00 a.m!", "I received two reminder calls at 1:30 a.m. and did not appreciate that", and "one time, I received a call at 11:00 p.m. and that made me think about dropping the program. That was not considerate." In addition, there were problems with system design: some participants were not happy with the frequency of calls received. In the open-ended interviews, participants reported that the system called to remind them regarding every pill, which for some was "too much" because they were on many medications.

Another major performance issue was that the voice recognition feature of IVR system had difficulty recognizing participants' voices. This caused much frustration for participants, who expressed that "the machine would not catch the yes or the no and sometimes would give an off response", "the machine made you sound stupid because it would ask you to answer even when you already had. It was confusing and would not listen to you and repeat the same thing", and that "it went very quick, did not give you any chances and would just bark something out at you."

### Acceptance of IVR technology

It was evident from the analysis of participants' comments from the open-ended questions that many participants were not receptive to the IVR technology. One reason was a frustration with not being able to talk to a real person: "I was irritated because I could not really speak to anybody and/or ask questions", "sometimes the calls were annoying and talking to a machine was not helpful", and "a machine is a machine. There is nothing human about that. I'd rather talk to a real person." In addition, some expressed discomfort with the technology in general, stating "it was intimidating because a computer was talking", "I don't need anything that's automatic", and that "the system was a failure and I had a terrible experience. The person who designed it had no humanistic understanding."

### IVR system utility

Twenty (71.4%) interview participants responded that they had already filled their medication prescription at the time they had received the IVR reminder call (Table 2). This issue was also identified by comments participants made during the follow-up interview. Participants stated "I don't need to be reminded, I am not senile yet", "we only have a certain number of pills to take and we remember them", and "it (the IVR system) does not do anything for me. It is useless because I take care of it myself. When there are only a couple of pills left, I go to the pharmacy. It is part of my routine." The general attitude of most participants was that they did not have trouble remembering to take medications or refill medication prescriptions. Only 4 participants (14.3%) responded that they found the system helpful for remembering to refill their prescriptions. However, many did indicate that this intervention could be helpful for those who do have difficulties remembering, stating "it is not useful now, maybe later or for others", and that the IVR system "seemed like a good idea for others but not for me."

### Comparing those who found the IVR system helpful with those who did not

In an attempt to identify characteristics of those who would find an IVR system most useful for remembering to take medications or refill prescriptions, those who responded that they found the system helpful for remembering to refill prescriptions were compared to those who responded that they did not find the system helpful Although significant differences between the two groups could not be determined due to the small sample size, there appeared to be a trend that participants who found the system helpful were more likely to be older and to have more active prescriptions. In addition, all four participants who found the system helpful understood that the IVR calls were to remind them to take or refill medication the first time the system called, whereas only 9 of the 24 who did not find the system helpful understood the purpose of the first IVR calls. Both groups encountered technical difficulties and expressed frustration with the IVR system in the open-ended interview, but those who found the system helpful for remembering to refill prescriptions made comments such as "(the IVR system) is a good idea, not because I forget but because it is good to be reminded" and "moderately (helpful), keeps track of pills", and also reported fewer negative comments overall than those who did not find the IVR system helpful.

## Discussion

IVR systems have the potential to enhance medication adherence for chronic conditions by providing medication and prescription refill reminders. However, this study identified difficulties in successfully implementing an IVR system for refill reminders in a population of persons taking medications for chronic diseases.

In order to use IVR systems efficiently, the population targeted for the intervention must be one where there is sufficient opportunity for the intervention to have an effect. In this study all persons on chronic disease-related medications were targeted for participation, but the results of the structured survey and open-ended questions indicated that this population may not have been in need of IVR medication reminders. Most participants responded that they did not have difficulties remembering to take medications or to get prescriptions refilled; in other words, they were still able to manage their medications independently. As a result, only four of the 28 participants who were interviewed and received reminder calls found the system helpful. In order to use IVR systems effectively as medication reminder systems in future studies, only those already having medication compliance problems due to forgetting to take or refill medications should be targeted for receiving the IVR system intervention.

Some of the technical and system design problems encountered could be addressed easily with system adjustments. For example, the frequency with which the IVR system was calling participants could easily be changed by adjusting the system so that it would recognize when multiple prescriptions for the same person needed to be refilled within a short time period and to call only once for all mediations. In addition, creating an option for participants to be called only for medications selected by either the patient or his/her physician could help resolve this issue.

The other major technical problem was the difficulty the voice response technology had recognizing participants' voices. The investigators discovered that the system had trouble recognizing a participant's voice if they were responding on a cordless phone but had created their voiceprint on a land line, or vice-versa. A simple solution to this problem would be to emphasize to participants to complete the voiceprint on the phone they are most likely to answer. In addition, the system often incorrectly recognized AM as PM and or PM as AM, and therefore called participants at inappropriate times. To solve all voice recognition problems key-pad response ability could be added to the system for times when the voice recognition fails. However, this doesn't address the goal of increasing accessibility of the system for those with limited manual dexterity or visual impairment. The best solution would be an improvement in speech recognition technology before re-piloting this type of intervention, which has been the case in recent IVR industry trends; speech recognition technology has improved greatly, now showing speech recognition accuracy rates between 90%–96% [23].

Issues such as resistance to IVR technology may limit the utility of this technology for many patients. Participants' feedback indicated that they found talking to an automated voice impersonal and awkward. This raises the question of the acceptability in general of health-related interventions that involve speaking to an automated voice, even with improvements in voice recognition technology and targeting the intervention to a population showing the greatest opportunity for the intervention to have an effect. Adding the option of key-pad responses may improve this acceptability. In addition, identifying characteristics of those who are more receptive to IVR technology and targeting this group is vital to effective implementation of IVR systems.

One limitation of this study was the small sample size. It is known that those who consent to participate in health research are often healthier than those who refuse. In this case, those who participated would be more likely to comply with their medication regimes, leading to an underestimation of the effectiveness of the intervention. On the other-hand, this self-selected group may also be more open to new technologies than those who refuse to participate, leading to an underestimation of the negative reaction found in this study to IVR technology. Therefore, although negative perceptions of the technology may be underestimated, the usefulness of the intervention may also be underestimated.

With only 38 people participating in the follow-up interview and only four responding that they thought the system was helpful, results relating to identifying characteristics of those receptive to IVR technology can be considered as trends to be investigated in future research; we cannot draw conclusions about the best target population for this intervention. However, it is interesting to note that all of the participants who reported the system as helpful understood what the IVR system was calling about the first time they received a call, whereas the majority of those who did not find the system helpful did not understand the purpose of the first IVR system phone call. If the amount of confusion regarding the IVR system phone calls could be reduced, more participants would find the system helpful. To reduce confusion, the initial IVR system call to set up individual preferences and voiceprints could be completed immediately after consent, followed by a call from a health team member to address any problems or questions about the set-up. An option to "zero out" (dial zero) during the phone call in order to reach a health team member could also help address confusion and questions during any IVR system call. Nevertheless, future research should attempt to identify more characteristics of those receptive to IVR technology.

As demonstrated, there were many technical issues discovered during implementation, even though the IVR system had been tested in-house by research staff multiple times before initiating the pilot study. This made it difficult to differentiate participant frustration regarding the technical functioning of the IVR system from participant resistance to the IVR system itself. Having a technically flawless system is therefore of utmost importance in determining the true ability of participants to accept an IVR system intervention.

## Conclusion

The goal of this pilot study was to evaluate the feasibility and acceptability of an IVR system for prescription refill and daily medication reminders in an ambulatory, primary care setting that provided two features not previously tested: medication-specific reminders for participants following real medication regimens, and communication via voice recognition. The medication-specific reminders, although generally not perceived as being helpful for this pilot study's population, were thought to be potentially helpful for those currently having difficulties remembering to take or refill medications. The voice recognition technology did not function properly, and therefore it was difficult to determine if the negative reception of the technology by participants was mainly due to the many technical flaws, a dislike for the technology itself, or both. In order to improve the implementation, utility, and acceptance of IVR systems, they should have solid technical performance and system design, and be implemented in a population where there is opportunity for the intervention to have an effect and where there is a willingness to accept new technology. Once these criteria have been satisfied, IVR systems have the potential to find their niche in the health care system as efficient and effective tools for improving medication compliance through medication and prescription refill reminders.

## Competing interests

The authors declare that they have no competing interests.

## Authors' contributions

KR performed the analysis and drafted the manuscript. RT conceived of the study, its design, oversaw its coordination and helped to draft the manuscript. VP helped to complete the qualitative analysis and draft the manuscript. AH participated in the design and coordination of the study. All authors read and approved the final manuscript.

## Pre-publication history

The pre-publication history for this paper can be accessed here:


